# Identification of Genomic Regions Associated with Differences in Flowering Time and Inflorescence Architecture between *Melastoma candidum* and *M. normale*

**DOI:** 10.3390/ijms251910250

**Published:** 2024-09-24

**Authors:** Jingfang Chen, Yan Zhong, Peishan Zou, Jianzhong Ni, Ying Liu, Seping Dai, Renchao Zhou

**Affiliations:** 1 School of Life Sciences, State Key Laboratory of Biocontrol and Guangdong Provincial Key Laboratory of Plant Stress Biology, Sun Yat-sen University, Guangzhou 510275, China; chenjf87@mail2.sysu.edu.cn (J.C.); zhongy328@mail.sysu.edu.cn (Y.Z.); liuyng73@mail.sysu.edu.cn (Y.L.); 2 School of Ecology, Sun Yat-sen University, Guangzhou 510275, China; 3 Guangzhou Institute of Forestry and Landscape Architecture, Guangzhou Collaborative Innovation Center on Science-Tech of Ecology and Landscape, Guangzhou 510405, China; zoupsh@mail2.sysu.edu.cn (P.Z.); njz899@163.com (J.N.)

**Keywords:** floral traits, genetic basis, inversion, QTL-seq, species difference

## Abstract

Understanding the genetic basis of species differences in flowering time and inflorescence architecture can shed light on speciation and molecular breeding. *Melastoma* shows rapid speciation, with about 100 species formed in the past few million years, and, meanwhile, possesses high ornamental values. Two largely sympatric and closely related species of this genus, *M. candidum* and *M. normale*, differ markedly in flowering time and flower number per inflorescence. Here, we constructed an F2 population between *M. candidum* and *M. normale*, and used extreme bulks for flowering time and flower number per inflorescence in this population to identify genomic regions underlying the two traits. We found high differentiation on nearly the whole chromosome 7 plus a few regions on other chromosomes between the two extreme bulks for flowering time. Large chromosomal inversions on chromosome 7 between the two species, which contain flowering-related genes, can explain recombinational suppression on the chromosome. We identified 1872 genes with one or more highly differentiated SNPs between the two bulks for flowering time, including *CSTF77*, *FY*, *SPA3*, *CDF3*, *AGL8*, *AGL15*, *FHY1*, *COL9*, *CIB1*, *FKF1* and *FAR1*, known to be related to flowering. We also identified 680 genes with one or more highly differentiated SNPs between the two bulks for flower number per inflorescence, including *PNF*, *FIL* and *LAS*, knows to play important roles in inflorescence development. These large inversions on chromosome 7 prevent us from narrowing down the genomic region(s) associated with flowering time differences between the two species. Flower number per inflorescence in *Melastoma* appears to be controlled by multiple genes, without any gene of major effect. Our study indicates that large chromosomal inversions can hamper the identification of the genetic basis of important traits, and the inflorescence architecture of *Melastoma* species may have a complex genetic basis.

## 1. Introduction

Flowering, which is essential for plants to reproduce, has a pivotal role in the sexual reproduction and evolution of flowering plants. Flowering time differentiation between populations is considered as one of the most important means to trigger speciation and maintain species integrity in plants [1,2]. For example, speciation in diurnally and nocturnally flowering populations can be initiated by differences in flowering time [3], and the divergence between two wild rice species, *Oryza nivara* and *O. rufipogon*, is maintained mainly by flowering time difference [4]. Six genetic pathways of flowering time, namely, vernalization, photoperiod/circadian clock, ambient temperature, gibberellin, autonomous, and age pathways, have been comprehensively characterized in the model plant *Arabidopsis thaliana* [5,6]. However, we know little about the genetic basis of flowering time difference in other plants, particularly non-model plants.

Meanwhile, floral traits, such as flowering time and inflorescence architecture (like the number of flowers per inflorescence), are also critical components of economical and ornamental values in crops and horticultural plants. In some crops, such as wheat (*Triticum*), rice (*Oryza sativa*), and barley (*Hordeum vulgare*), the number of spikelets per spike and the number of fertile florets per spikelet mainly determine grain yield, with more yield for greater grain numbers per inflorescence [7]. In horticultural plants, more flowers per inflorescence usually have higher ornamental value and longer flowering time. Therefore, exploring the genetic basis of floral traits can provide insights into not only the evolution of reproductive isolation between closely related species, but also the breeding of new cultivars with more agricultural and horticultural values.

Identifying genomic regions and molecular markers associated with traits of interest is an important goal for studying trait evolution and molecular-assisted selection. Bulk segregation analysis (BSA) was applied to two bulked DNA samples with extreme phenotypes from a segregating population generated from two parents with different traits to develop allelic variation associated with the traits [8]. Compared with traditional QTL mapping, BSA is a shortcut to detect genetic differences between two populations without characterizing a large number of individuals, which dramatically reduces the costs, labor and space [9]. Next-generation sequencing (NGS)-based genotyping techniques dramatically increase the effectiveness and accuracy of BSA for identification of markers tightly linked to the causal gene for a given phenotype and QTL mapping. Up to now, NGS-based bulk segregation analyses have been widely applied in QTL mapping, and have discovered QTLs associated with agronomic traits, such as seed weight in chickpea (*Cicer arietinum*) [10], flowering time and leaf shape in pigeonpea (*Cajanus cajan*) [11], twig trichome color variation in *Melastoma normale* [12], flowering time, fruit flesh thickness, pre-harvest sprouting and downy mildew resistance in cucumber [13,14,15,16], cold tolerance, salt tolerance and blast resistance in rice [9,17,18], cotyledon color and nutritional ingredient in soybean [8,19], and so on.

*Melastoma* L., a shrub genus mainly distributed in tropical Asia and Oceania, consists of approximately 100 species [20,21]. It has undergone rapid species radiation within the past 4 million years [22], providing an excellent opportunity to study rapid speciation and the genetic basis of adaptive traits. Species of *Melastoma* also possess high ornamental values, and some cultivars have been bred by artificial interspecific crosses [23]. Two main species of this genus, *M. candidum* and *M. normale*, are both light-demanding opportunists and usually occur in open fields, grasslands and roadsides [24]. They are often sympatric in South China. Although their distribution and habitats are overlapped, no hybrids between them are observed in nature due to their different flowering time. In South China, *M. normale* typically flowers from late March to late April, while *M. candidum* flowers from early June to late July [24]. The difference in flowering time leads to the reproductive isolation of the two species, which should play an important role in maintaining their genetic integrity despite their being in sympatry. They also show marked differences in inflorescence architecture, especially the number of flowers per inflorescence, with, usually, more than nine (up to fifteen) flowers per inflorescence in *M. normale* and at most five flowers per inflorescence in *M. candidum*. The number of flowers per inflorescence in *Melastoma* is one of its most important ornamental traits, and it may be related to the efficiency of pollination and sexual reproduction [25]. Therefore, the two species provide ideal materials to investigate the genetic basis of these key traits in *Melastoma*, a non-model genus of woody plants.

Many floral traits, including flowering time, have a complex genetic architecture and are controlled by multiple genes [6]. Flowering time and inflorescence architecture vary greatly among species of *Melastoma*; however, their genetic basis remains unknown. In this study, an F2 segregating population from interspecific crosses between *M. candidum* and *M. normale* was constructed to identify genomic regions and candidate genes associated with flowering time and inflorescence architecture (flower number per inflorescence), using bulk segregation analysis based on Illumina sequencing. Understanding species differences in the floral characteristics of *Melastoma* may not only shed light on the mechanisms of reproductive isolation and floral evolution, but also contribute to molecular-assisted selection of new cultivars in this genus.

## 2. Results

### 2.1. Highly Differentiated Regions and SNPs between the Two Extreme Bulks for Flowering Time

**Figure 1:**
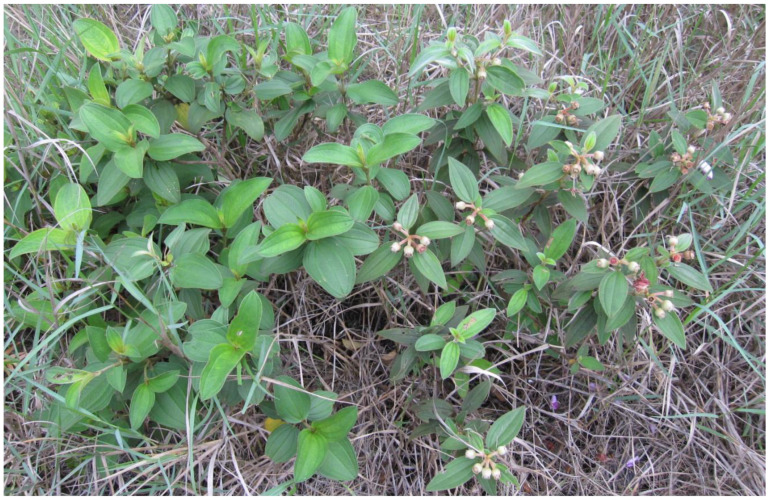
Flowering time difference between *Melastoma candidum* (left) and *M. normale* (right). The two species are largely sympatrical in South China. *Melastoma normale* typically flowers from late March to late April, much earlier than *M. candidum*, which flowers from early June to late July. This photo was taken on the Zhuhai campus of Sun Yat-sen University, Zhuhai, Guangdong, China, on 11 May 2012, when *M. normale* had already set fruits, while *M. candidum* had not begun to flower.

As described in the Introduction, the flowering time of *M. normale* is much earlier than that of *M. candidum* (Figure 1), and there is an interval of about one month between the end of flowering of *M. normale* (late April) and the beginning of flowering of *M. candidum* (early June). The earliest and latest flowering times in the F2 population are 4 April and 23 June, respectively. Calculated from January 1, days to flowering in this population range from 93 to 173. Days to flowering for the selected early flowering and later flowering bulks range from 93 to 111 and from 155 to 173, respectively.

**Figure 2:**
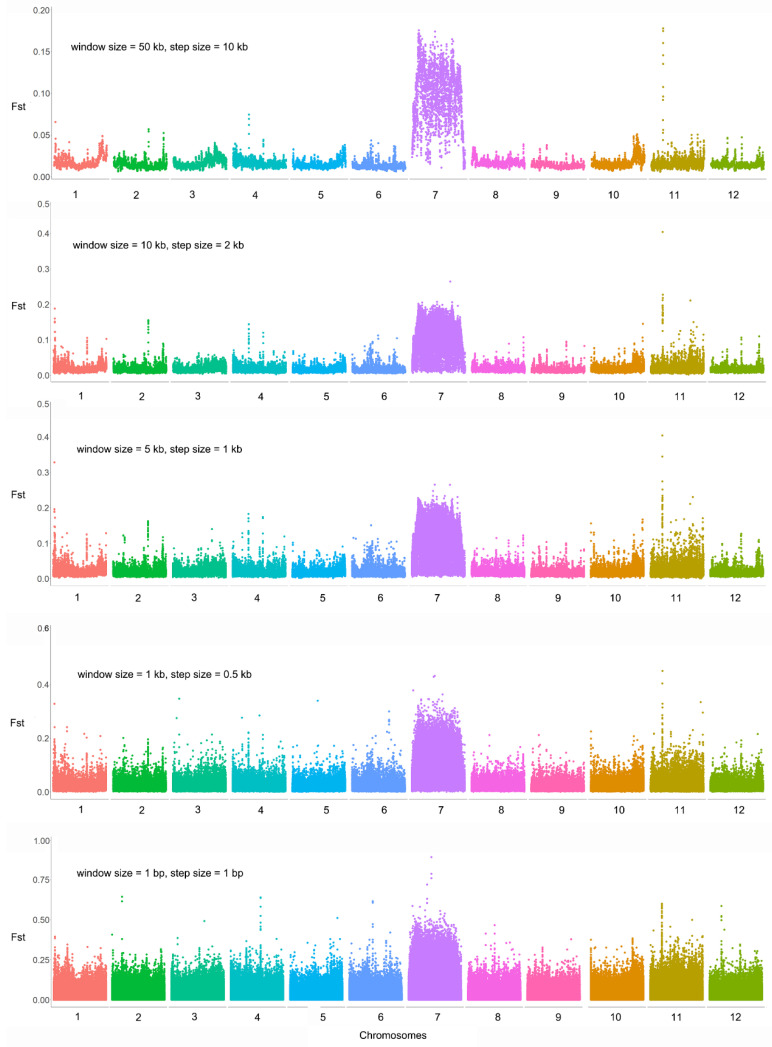
Genomic differentiation between two extreme bulks for flowering time in an F2 population between *Melastoma candidum* and *M. normale*. Sliding window Fst analyses were conducted with five sets of different window size and step size, as shown in the upper left of each panel.

**Figure 3:**
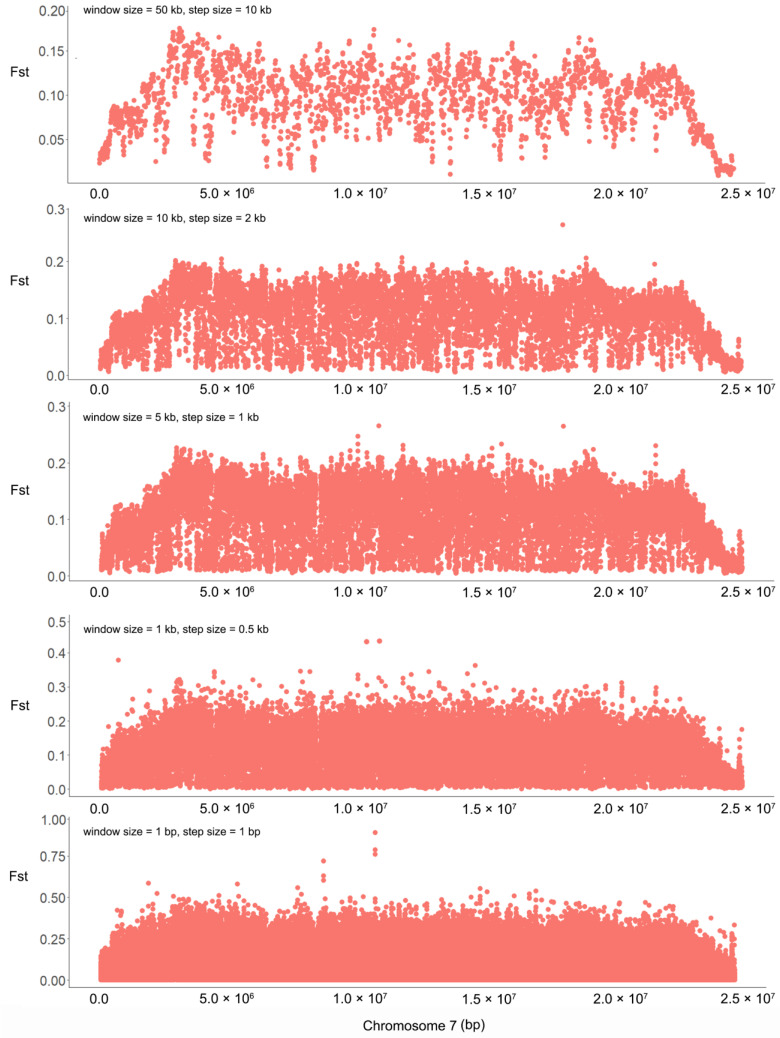
Genetic differentiation on chromosome 7 between two extreme bulks for flowering time in an F2 population between *Melastoma candidum* and *M. normale*. Sliding window Fst analyses were conducted with five sets of different window sizes and step sizes, as shown in the upper left of each panel.

After mapping Illumina reads of the two extreme bulks for flowering time to the *M. candidum* reference genome, a total of 7,996,211 SNPs were identified on the 12 chromosomes for the two bulks, with SNP densities ranging from 28.03 to 41.32 SNPs/kb across the 12 chromosomes. Genetic differentiation between the two bulks in the F2 population is generally low across the genome (average Fst = 0.019 based on all SNPs across the genome) except for chromosome 7 (average Fst = 0.057 based on 701,408 SNPs on this chromosome) (Figure 2, Table S1). It is surprising that nearly the whole of chromosome 7 shows high differentiation between the two extreme bulks, regardless of the window size we used in the sliding-window Fst analysis (Figure 2 and Figure 3). Across the genome, there are 4476 SNPs with an Fst value > 0.3, among which there are 49 SNPs with an Fst value > 0.5 (the maximum Fst = 0.89) and 312 SNPs with an Fst value of 0.4–0.5. Among the 4476, 312 and 49 SNPs, 4168 (93.12%), 284 (91.03%) and 24 (48.98%) were located on chromosome 7 (Figure 3), indicating that most highly differentiated SNPs concentrate on this chromosome.

**Figure 4:**
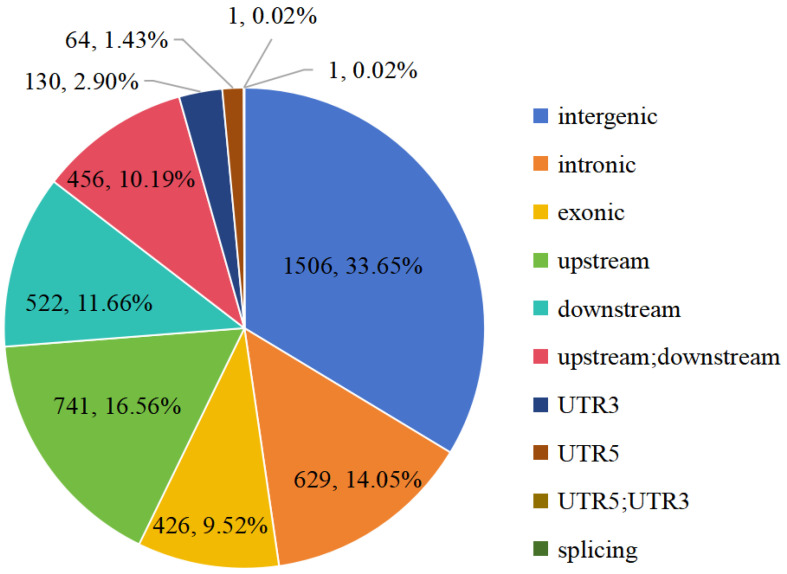
Genomic location classification of highly differentiated (Fst > 0.3) SNPs between two extreme bulks for flowering time in an F2 population between *Melastoma candidum* and *M. normale*. The numbers of SNPs and their percentages are shown. Exonic: SNPs occurring in a coding region; intronic: SNPs occurring in an intron; splicing: SNPs occurring within 2 bp near the exon/intron boundary in an intron; upstream: SNPs occurring in 2 kb upstream of transcription start site; downstream: SNPs occurring in 2 kb downstream of transcription end site; upstream;downstream: SNPs occurring in 2 kb upstream of transcription start site of a gene and, meanwhile, 2 kb downstream of transcription end site of another gene; intergenic: SNPs occurring in intergenic region; UTR5: SNPs occurring in a 5′ untranslated region; UTR3: SNPs occurring in a 3′ untranslated region; UTR5; UTR3: SNPs occurring in a 5′ untranslated region of one gene and, meanwhile, a 3′ untranslated region of another gene.

The genomic location classification of the 4476 highly differentiated SNPs is shown in Figure 4. Most of these SNPs are located in intergenic spacers, and only 426 (9.52%) and 629 (14.05%) of them were found in exonic and intronic regions, respectively. The 4476 SNPs occur in 1872 genes, among which 1685 (90.01%) were located on chromosome 7. Among the 1872 genes, 1497 (79.97%) were functionally annotated by searching against the Swiss-Prot database, 1263 (67.47%) were assigned to GO terms of biological process and 412 (22.01%) were assigned to the KEGG pathways. Only one significantly enriched GO term, nucleosome assembly (GO:0006334, corrected P ≈ 0.005), was found (Table S2). No significantly enriched KEGG pathways were found (Table S3).

The 292 flowering time-related genes in *Arabidopsis thaliana* summarized in previous studies [26,27] correspond to 367 orthologous genes in *M. candidum* (Table S4), suggesting there is *M. candidum*-specific duplication of some genes, which is consistent with the existence of two rounds of whole genome duplications in *Melastoma* after it diverges from *Eucalyptus* [22]. Of the 367 genes, 37 are located on chromosome 7, suggesting no enrichment of flowering time-related genes on chromosome 7. Of the 367 genes, 15, namely *CSTF77*, *FY*, *SPA3*, *CDF3*, *AGL8*, *FUS3*, *AGL15*, *FHY1*, *EZA1*, *COL9*, *CIB1*, *FKF1*, *FAR1*, *PGM* and *HAP2A*, contain one or more highly differentiated SNPs, and are all located on chromosome 7 (Table 1). Among the genes in the six pathways of flowering time in *Arabidopsis*, four orthologous genes in *Melastoma*, *AGL8* (belonging to the age pathway), *CDF3*, *CIB1* and *FKF1* (belonging to the photoperiod pathway), contain one or more highly differentiated SNPs (Table 1).

The SNP with the highest Fst value (0.89) was located on chromosome 7 and in the intergenic spacer between two *M. candidum* genes (Gene IDs MLD38_025448 and MLD38_025449), which encode G6PDC (glucose-6-phosphate 1-dehydrogenase, chloroplastic) and MAD57 (MADS-box transcription factor 57), respectively. *MAD57* in rice controls the outgrowth of axillary buds, and overexpression of *MADS57* can sustain rice tiller growth under cold stress [28,29], but it is unknown if this gene has a function in inflorescence development in *Melastoma*. The most highly differentiated region on chromosome 11 (see Figure 2) contains a gene (Gene ID MLD38_036042) encoding BCAL2 (branched-chain amino acid aminotransferase-like protein 2).

### 2.2. Large Inversions on Chromosome 7 between M. candidum and M. normale

**Figure 5:**
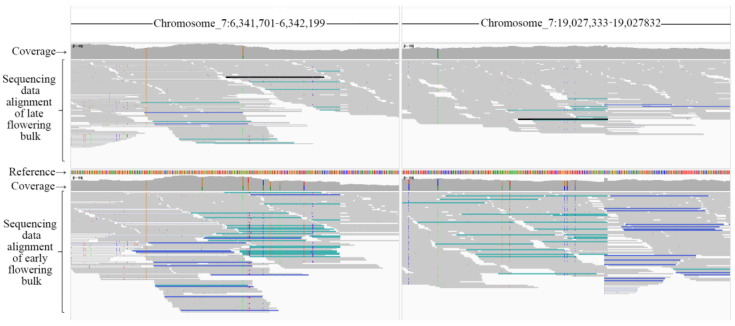
Visualization of the largest inversion on chromosome 7 detected from Illumina sequencing data mapping files of two extreme bulks for flowering time in an F2 population between *Melastoma candidum* and *M. normale* using Integrative Genomics Viewer. To visualize, we used the options of “set color alignments by pair orientation” and “view mate region in split screen”. Paired-end reads with blue and cyan support inversion. The region ranges containing the inversion breakpoints on chromosome 7 are indicated on the top.

The high differentiation on nearly the whole chromosome 7 between the two extreme bulks for flowering time is unusual in bulk segregation analysis, and this suggests that recombination on chromosome 7 between *M. candidum* and *M. normale* is highly suppressed. Because large chromosomal inversions can suppress recombination [30], we test if there is any large chromosomal inversion on chromosome 7 between the two species. Through mapping of Illumina sequencing data of the three F1 individuals on the *M. candidum* genome, we identified five inversions > 1 Mb in size on this chromosome, and all of them were supported by paired-end reads and split reads (Table 2, Figure S1). As one haplotype in the F1 individuals stems from *M. candidum* and the other from *M. normale*, inversions identified in the F1 individuals indicate that there exist inversions between the two species, although we do not know if these inversions are fixed in one species or not. These inversions were also identified in the two extreme bulks for flowering time from the F2 population. The longest inversion is 12.7 Mb, from the nucleotide position 6.3 Mb to 19.0 Mb on chromosome 7 (Figure 5), and the shortest inversion is about 1.8 Mb, from the nucleotide position 10.9 Mb to 12.7 Mb. These inversions span 18.3 Mb (from the nucleotide position 0.7 Mb to 19.0 Mb) of chromosome 7, covering 74.7% of the 24.5 Mb chromosome 7. All the inversion regions contain one or more genes known to be related to flowering in *Arabidopsis*.

### 2.3. Highly Differentiated Regions and SNPs between the Two Extreme Bulks for Flower Number per Inflorescence

**Figure 6:**
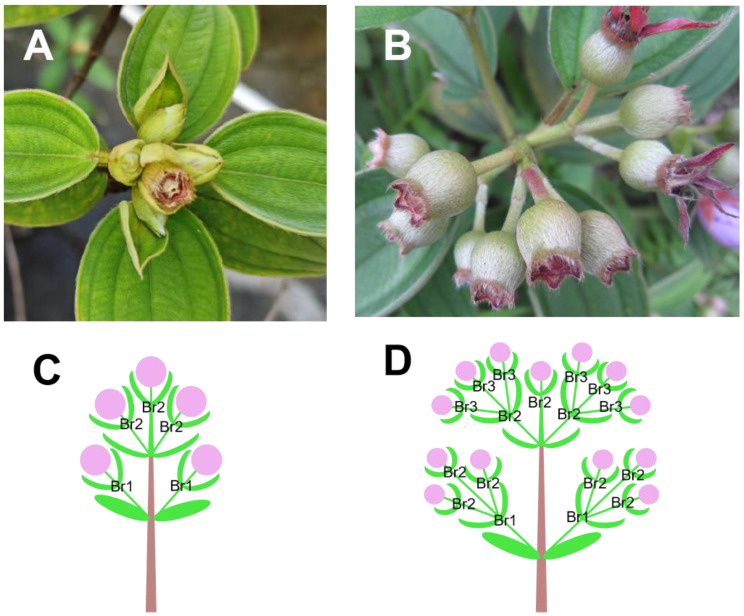
Differences in inflorescence architecture (flower number per inflorescence) between *Melastoma candidum* and *M. normale*. (**A**) One fruit and four unopen flowers in an inflorescence of *M. candidum*; (**B**) the fruits in an infructescence of *M. normale* (we show the fruits rather than the flowers because we could not provide photos to show clear inflorescence architecture when flowering). (**C**) A schematic diagram of the inflorescence architecture of *M. candidum*; (**D**) a schematic diagram of the inflorescence architecture of *M. normale*. (**C**,**D**) Purple filled circles, green crescents and green ovals represent flowers, bracts and leaves, respectively, and Br1, Br2 and Br3 represent the first-, second- and third-order branches in the inflorescence.

Both *M. candidum* and *M. normale* have terminal, dichasial cymes, but they differ in flower number per inflorescence: *M. candidum* usually has 3–5 flowers per inflorescence, while *M. normale* usually has >9 flowers per inflorescence (Figure 6A,B). More flowers per inflorescence in *M. normale* result from more inflorescence branching on the rachis, with only first-order branches (three flowers) and first- and second-order branches (five flowers) in the inflorescence of *M. candidum*, and first-, second- and third-order branches in that of *M. normale* (Figure 6C,D). The flower number per inflorescence in the F2 population ranges from 3.3 to 11.3. The flower number per inflorescence for the selected high flower number per inflorescence and the lower flower number per inflorescence bulk range from 3.3 to 4.3 and from 8.0 to 11.3, respectively.

**Figure 7:**
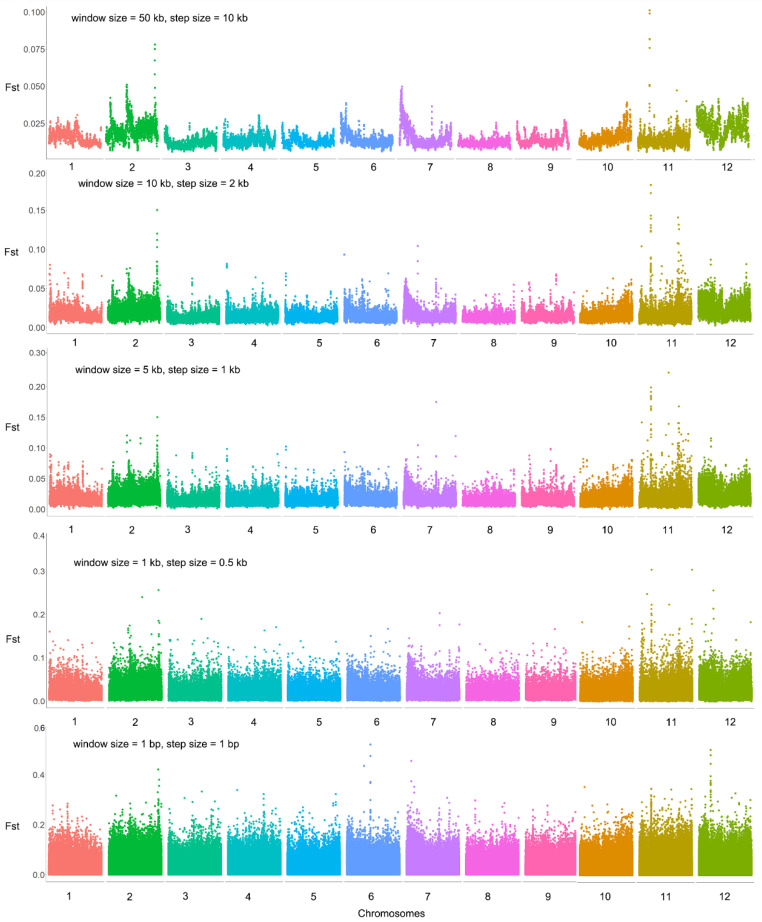
Genomic differentiation between two extreme bulks for flower number per inflorescence in an F2 population between *Melastoma candidum* and *M. normale*. Sliding window Fst analyses were conducted with five sets of different window sizes and step sizes, as shown in the upper left of each panel.

After mapping Illumina reads of the two extreme bulks for flower number per inflorescence to the *M. candidum* reference genome, a total of 7,673,521 SNPs were identified on the 12 chromosomes for the two bulks, with SNP densities ranging from 27.3 to 39.9 SNPs/kb across the 12 chromosomes. Again, sliding-window Fst analyses with different window and step sizes showed that genetic differentiation between the two bulks in the F2 population is generally low across the genome (average Fst = 0.014 based on all SNPs across the genome). Unlike the two extreme bulks for flowering time, which show high differentiation on nearly the whole chromosome 7, the two extreme bulks for flower number per inflorescence exhibit only a few highly differentiated regions on several chromosomes (Figure 7, Table S1). Across the genome, there are 759 SNPs with an Fst value > 0.2, among which there are 3 SNPs with an Fst value > 0.5 (the maximum Fst = 0.569), 6 SNPs with an Fst value of 0.4–0.5, 45 SNPs with an Fst value of 0.3–0.4 and 705 SNPs with an Fst value of 0.2–0.3.

**Figure 8:**
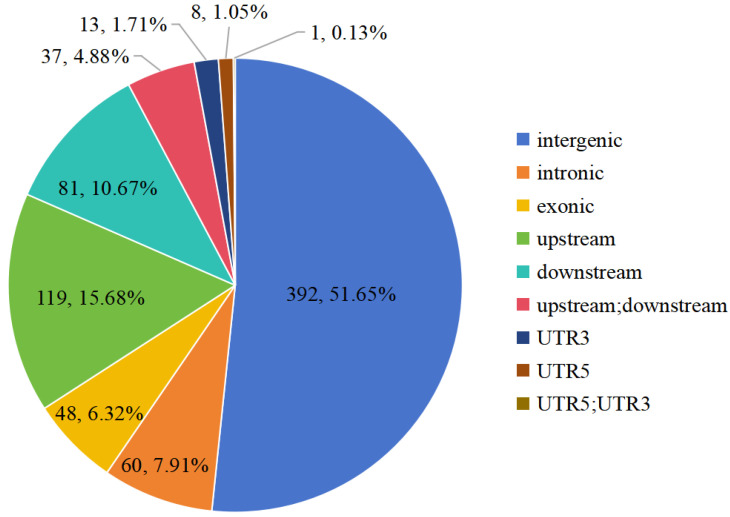
Genomic location classification of highly differentiated (Fst > 0.2) SNPs between two extreme bulks for flower number per inflorescence in an F2 population between *Melastoma candidum* and *M. normale*. See Figure 4 for the legend of each category.

The genomic location classification of 759 highly differentiated SNPs is shown in Figure 8. Most of these SNPs are located in intergenic spacers, and only 48 (6.32%) and 60 (7.91%) of them were found in exonic and intronic regions, respectively. The 759 SNPs were assigned to 680 genes, among which 489 (71.91%) genes were functionally annotated by searching against the Swiss-Prot database, 387 (56.91%) genes were assigned to GO terms of biological process and 115 (16,91%) genes were assigned to the KEGG pathways. Two significantly enriched GO terms, DNA integration (GO:0015074) and proteolysis (GO:0006508), were found (Table S5), but they are not related to flowering. Also, no significantly enriched KEGG pathways were found (Table S6). The 66 inflorescence architecture-related genes in *A. thaliana* and other species correspond to 100 orthologous genes in *M. candidum* (Table S7), again suggesting there is *M. candidum*-specific duplication of some genes. Four of the 100 genes, namely *JAG*, *PNF*, *FIL* and *LAS*, contain one or more highly differentiated SNPs (Table 3).

The SNP with the highest Fst value (0.57) was located in the intergenic spacer of two *M. candidum* genes (Gene IDs MLD38_038216 and MLD38_038217) on chromosome 12, which encode NF-YC11 (nuclear factor Y, subunit C11) and WTR38 (WAT1-related protein), respectively. The SNP with the second highest Fst value (0.52) was located in the intergenic spacer of two genes (Gene IDs MLD38_021340 and MLD38_021341) on chromosome 6, which encode HCAR (7-hydroxymethyl chlorophyll a reductase, chloroplastic) and HGL1 (heteroglycan glucosidase 1), respectively. The most highly differentiated region on chromosome 11 (see Figure 7) contains one gene (Gene ID MLD38_036043) encoding BCAL2 (branched-chain amino acid aminotransferase-like protein 2).

## 3. Discussion

### 3.1. Genomic Regions Related to Flowering Time in Melastoma

Our BSA-seq revealed that nearly the whole chromosome 7, plus a few regions on other chromosomes, show high differentiation between the two extreme bulks for flowering time. It is surprising that nearly the whole chromosome shows high differentiation between the two bulks, because meiotic recombination will cause allelic exchanges between homologous chromosomes except the genomic regions related to flowering time we selected for the two extreme bulks. There are two possible explanations for the chromosome-scale high differentiation. One explanation is the enrichment of most flowering time-related genes on nearly the whole chromosome 7, and these genes collectively determine the flowering time difference between *M. candidum* and *M. normale*. However, no enriched GO terms and KEGG pathways related to flowering were found in the GO and KEGG enrichment analysis for highly differentiated genes (most on chromosome 7). Furthermore, of 367 *M. candidum* orthologs of flowering-related genes in *Arabidopsis*, only 37 (10.1%) were located on chromosome 7. So, this explanation does not hold. The other explanation is that large inversion(s) exists on chromosome 7 between *M. candidum* and *M. normale*, and, meanwhile, the inversion(s) contains one or more genes contributing mainly to flowering time differences between the two species, because large inversion(s) can highly suppress recombination [31,32,33,34]. Our results are consistent with this explanation. On one hand, we identified five large inversions on chromosome 7, including the largest one of 12.7 Mb, which collectively span nearly the whole chromosome. On the other hand, the highly differentiated regions on chromosome 7 indeed contain a few genes related to flowering time (Table 2).

Among 15 candidate flowering time-related genes, *CDF3*, *CIB1* and *FKF1* genes are included in the photoperiod pathway that controls flowering in *Arabidopsis.* CDF3 (Cyclic DOF factor 3) is one of the finger (DOF)-type transcription factors that are implicated in responses to light and phytohormones, and repress flowering by downregulating *CONSTANS* (*CO*), a gene that accelerates flowering in response to long days [6,35]. CIB1 (cryptochrome-interacting basic helix-loop-helix) interacts with CRY2 (a cryptochrome that mediates light response) and CIB1-related proteins to promote CRY2-dependent floral initiation [36]. FKF1 (FLAVIN-BINDING, KELCH REPEAT, F-BOX 1) controls the daytime expression pattern of *CO* [37,38]. *AGL8*, also known as *FRUITFULL* (*FUL*), belongs to the age pathway in *Arabidopsis*, and regulates FLOWERING LOCUS C (FLC) [39], the key gene in both the vernalization pathway and the autonomous flowering pathway [40]. *FY*-encoding protein interacts with FLC and FCA, with the latter being a post-transcriptional regulator that strongly promotes the transition to flowering in *Arabidopsis* [41]. The interactions of FY, FCA and FLC regulate flowering time through the autonomous pathway in *Arabidopsis* [42]. CSTF77 (cleavage stimulation factor subunit 77) is a conserved RNA 3′-end-processing factor, which is required in *FLC* silencing and, thus, regulates the onset of flowering [43]. *SPA3* (*SPA1-related 3*), a member of the *Suppressor of phyA-105* (*SPA1*) gene family, regulates photomorphogenesis in the light in *Arabidopsis* seedlings [44]. *COL9* is a member of the *CONSTANS-like* (*COL*) gene family, and overexpression of *COL9* results in delayed flowering in transgenic *Arabidopsis* [45]. AGL15 acts on the upstream of *FLOWERING LOCUS T* (*FT*), and increased expression of *FT* can delay flowering time [46,47]. FHY1 (FAR-RED ELONGATED HYPOCOTYL 1), a member of the photoperiod pathway and primarily involved in far-red light sensing, mediates nuclear accumulation of the Phytochrome A (PHYA) in *Arabidopsis* [48,49]. NFYA1 (nuclear transcription factor Y subunit A-1) is a subunit of the HAP complex, and the expression of HAP complex subunits affects flowering time by reducing activation of FT in *Arabidopsis*. In summary, the *M. candidum* orthologs of these flowering-related genes contain highly differentiated SNP(s), suggesting that they might contribute to flowering time differences between *M. candidum* and *M. normale*.

Large chromosomal inversions, which can severely inhibit recombination at the inversion regions, play important roles in reproductive isolation and local adaptation [31,50]. For example, 21 polymorphic inversions likely facilitate local adaptation, and cause near-complete suppression of recombination when heterozygous in deer mice [51]. An inversion of 115 Mb was discovered in the genome of the common quail, and individuals with this inversion are larger and have darker throat coloration and rounder wings, which cause poorer flight efficiency [52]. In the stick insect, a chromosomal inversion suppresses effective recombination and is associated with crypsis, which contributes largely to adaptation to different microhabitats [53]. A major inversion between subspecies of *Boechera stricta* contains genes controlling ecologically important traits, including flowering time difference, which is expected to enhance reproductive isolation between subspecies [54]. Locally adapted alleles can be captured by inversions and promote incipient speciation [34]. It is likely that a few flowering-related genes were captured by large inversions on chromosome 7 in *Melastoma*, which causes high differentiation on nearly the whole chromosome between the two extreme bulks for flowering time.

However, we could not determine which gene(s) confer flowering time differences between *M. candidum* and *M. normale*. Due to the presence of large inversions, it is possible that even variation in one causal gene related to flowering time within an inversion can cause high differentiation in the whole region spanning the inversion between the two extreme bulks for flowering time. So, we could not narrow down the region associated with flowering time within the inversion.

### 3.2. Genomic Regions Related to Flower Number per Inflorescence in Melastoma

All species of *Melastoma* have terminal, dichasial cymes, and most species, including *M. candidum,* usually have 3–5 flowers per inflorescence, while several species, including *M. normale*, *M. imbricatum*, *M. joffrei* and *M. malabathricum,* usually have >9 flowers per inflorescence [24]. As shown in Results, more flowers per inflorescence result from more inflorescence branching on the rachis.

Among candidate genes related to inflorescence architecture identified in other species, *PNF* is expressed in the shoot apical meristem in *Arabidopsis* and is required for the maintenance of boundaries between the initiating floral primordia and inflorescence meristem [55]; *FIL* (*FILAMENTOUS FLOWER*) regulates the inflorescence meristem and floral meristem development, and the *fil* mutant forms two types of inflorescences and flowers [56]. *LAS* is implicated in the process of axillary meristem initiation in *Arabidopsis* [57]. *NF-YC11* is expressed in vascular tissues and is a potential target for flowering time control in Arabidopsis [58], and its role in inflorescence development remains unclear. The *BCAL2* gene is an uncharacterized aminotransferase-like sequence [59]. As this gene also shows high differentiation between the two bulks for flowering time, we infer that this gene may have dual roles. A previous study also showed that some genes related to flowering also have functions in inflorescence development [6]. These genes with highly differentiated SNPs between the two bulks exist in different chromosomes, suggesting that flower number per inflorescence in *Melastoma* might be controlled by multiple genes, without any gene of major effect. The divergence of these genes between *M. candidum* and *M. normale* may result in differences in flower number per inflorescence between them. This can be tested in other species of *Melastoma* that also show differences in this trait.

In dogwoods (*Cornus*), expressional alternations of two inflorescence architecture genes (*TFL1* and *AP1*) among different species correlate with major evolutionary shifts in inflorescence architecture [60], and we speculate that difference in flower number per inflorescence among *Melastoma* species may also be associated with expressional changes in some inflorescence architecture genes identified in this study, which needs further testing. If so, molecular-assisted selection can be used for new cultivar breeding by examining the expressional patterns of these genes.

## 4. Materials and Methods

### 4.1. Construction of an F2 Segregating Population and Bulk Sampling

One individual each of *M. normale* and *M. candidum* was collected from Zhuhai, Guangdong and Shanglin, Guangxi, respectively, and both of them were planted on the campus of Sun Yat-sen University, Guangzhou. The pollens of *M. normale* were collected in March and stored immediately at −80 °C until the flowering of *M. candidum* in early June 2018. Stamens of *M. candidum* were removed one day before flowering, and pollens of *M. normale* were placed on the stigma of *M. candidum* on the first day of flowering. Pollinated flowers were bagged for two days until the styles fell. When the fruits ripened, F1 seeds were obtained and sowed. F2 hybrids were obtained through F1 selfing when F1 individuals started to flower in 2020. The F2 individuals were grown in 10 cm × 10 cm plastic pots in the greenhouse of Sun Yat-sen University under natural light conditions. Finally, an F2 segregating population comprising 188 individuals was obtained and used in the subsequent analysis. For each F2 individual, the flowering date of the first flower and flower number per inflorescence (we used the average number from three randomly chosen inflorescences) were recorded in 2022.

Extreme bulks for flowering time and flower number per inflorescence from the F2 population were sampled separately. Fresh leaves of 30 individuals each, with the earliest flowering, the latest flowering, the highest flower number per inflorescence and the lowest flower number per inflorescence, were collected, respectively. The leaves were dried in plastic bags with silica gels. For each of the four extreme bulks mentioned above, we pooled the dried leaf tissues with equal weights of all 30 individuals for DNA isolation, as conducted in [61]. In addition, we also collected fresh leaves of three F1 individuals and dried them using the same method.

### 4.2. Library Preparation and Illumina Sequencing

We extracted DNA from the four pooled samples and three F1 individuals using a Magen HiPure Plant DNA Mini Kit (Magen Biotech, Guangzhou, China). The quality and quantity of genomic DNA were determined by 1% agarose gel electrophoresis and a Qubit 3.0 Fluorometer (Invitrogen, Carlsbad, CA, USA). For each DNA sample, a sequencing library with an insert size of 400 bp was constructed using an Illumina TruSeq Nano DNA Library Prep Kit (Illumina, San Diego, CA, USA). The resulting four DNA libraries were sequenced on an Illumina HiSeq X Ten platform, generating about 30 Gb (~117 × of the *M. candidum* genome at 256.2 Mb) of paired-end raw reads of 150 bp (PE-150) for each sample. For the three F1 individuals, about 6.1, 6.7 and 7.1 Gb of PE-150 were generated, respectively.

### 4.3. Bulk Segregation Analysis

The raw reads were filtered using Trimmomatic (version 0.39) [62] with the parameters “ILLUMINACLIP:TruSeq3-PE:2:30:10 LEADING:3 TRAILING:3 SLIDINGWINDOW:4:15 MINLEN:100”. Clean reads of each sample were mapped to the reference genome of *M. candidum* (GenBank accession: GCA_023653495.1) using BWA-MEM [63] with default parameters. The aligned reads were sorted using the *sort* module and the PCR duplications were removed using the *rmdup* module in SAMtools version 1.10 [64]. Final alignment files for each extreme bulk pair were combined to generate multiple pileup files using the *mpileup* module in SAMtools.

The multiple pileup files were supplied to Popoolation2 [65] for analyzing the genetic differentiation between each of the two extreme bulk pairs. The pileup file for each bulk pair was synchronized using the *mpileup2sync.jar* module in Popoolation2. To reduce the influence of insertions/deletions (indels) on SNP identification, the two perl scripts *identify-indel-regions.pl* and *filter-sync-by-gtf.pl* in Popoolation2 were used to filter out regions around indels with default parameters. The Fst for each SNP was calculated using the script *fst-sliding.pl* in Popoolation2 with parameters of suppress-noninformative, min-count 2, min-coverage 20, max-coverage 2%, min-covered-fraction 1 and pool-size 30 and five different sets of window size and step size (window-size 50 kb, step-size 10 kb; window-size 10 kb, step-size 2 kb; window-size 5 kb, step-size 1 kb; window-size 1 kb, step-size 0.5 kb; and window-size 1 bp, step-size 1 bp).

### 4.4. Annotation of Highly Differentiated SNPs between Each Bulk Pair

The gene annotation file of the *M. candidum* genome was downloaded from the NCBI database, and converted to a GenePred file using the gtfToGenePred tool (https://hgdownload.cse.ucsc.edu/admin/exe/linux.x86_64/gff3ToGenePred; accessed on 13 March 2018). Then, the mRNA sequences were retrieved using the perl script *retrieve_seq_from_fasta.pl* in ANNOVAR version 2020-06-07 [66], and SNPs showing high genetic differentiation between each bulk pair were annotated using the script *annotate_variation.pl* with the parameter --neargene 2000 to identify genes containing these SNPs.

### 4.5. GO and KEGG Pathway Enrichment Analyses of Candidate Genes Associated with Flowering Time and Flower Number per Inflorescence

The identified genes were functionally annotated using BLAST 2.2.24+ (ftp://ftp.ncbi.nlm.nih.gov/blast/executables/blast+/LATEST/; accessed on 9 September 2011) against the Swiss-Prot database (https://ftp.ncbi.nlm.nih.gov/blast/db/FASTA/swissprot.gz; accessed on 6 February 2024). The Gene Ontology (GO) and KEGG pathway enrichment analyses of these candidate genes were carried out with the functional enrichment analysis tool DAVID [67], with all the *M. candidum* genes as the background.

### 4.6. Detection of Inversions between the Genomes of M. candidun and M. normale

Delly v.1.2.6 [68] was applied to detect inversions between the genomes of *M. candidun* and *M. normale* by analyzing Illumina sequencing data of three F1 individuals and two extreme bulks for flowering time, respectively. Briefly, Illumina sequencing data of the three F1 individuals were mapped to the reference genome of *M. candidum* using the same method described before. Then, the alignment file of each individual was used as input to call structural variants using Delly with default parameters. For an inversion between the genomes of *M. candidun* and *M. normale*, it is expected that one of the two alleles in the F1 individuals should carry the inversion. Therefore, we kept only inversions supported by about half of the paired-end reads and split reads on the mapped position. We then examined whether these inversions also exist in the two extreme bulks for flowering time. These inversions were further visualized and verified in Integrative Genomics Viewer (IGV) v. 2.16.2 [69].

### 4.7. Identification of Orthologous Proteins between Arabidopsis thaliana and M. candidum

The protein sequences of *A. thaliana* were downloaded from the TAIR website (https://www.arabidopsis.org/download/file?path=Proteins/Araport11_protein_lists/Araport11_pep_20220914_representative_gene_model.gz; accessed on 14 September 2022). Orthogroups of proteins between *A. thaliana* and *M. candidum* were identified using OrthoFinder v2.4.0 [70]. Orthogroups containing 292 flowering time genes in *A. thaliana* summarized in previous studies [26,27] were extracted (Table S4), and the corresponding orthologs in *M. candidum* were identified. Genes involved in the regulation of inflorescence architecture in *A. thaliana* and other species summarized from previous studies [71,72,73,74] were extracted (Table S7).

## 5. Conclusions

Using bulk segregation analyses, we identified genomic regions and genes associated with differences in flowering time and flower number per inflorescence between two *Melastoma* species. Surprisingly, nearly the whole of chromosome 7 shows high differentiation between early and late flowering time bulks in the F2 population. A gene or multiple genes related to flowering time difference within large chromosomal inversions on this chromosome between the two species should cause this chromosome-scale differentiation. These large inversions also prevent us from narrowing down the genomic region(s) associated with flowering time differences between the two species. Further studies can select other *Melastoma* species differing in flowering time but lacking in large inversions between them to identify genomic regions and genes underlying this trait. Flower number per inflorescence in *Melastoma* appears to be controlled by multiple genes without any gene of major effect. It remains unknown whether the expressional alternations of these identified genes correlate with differences in the number of flowers per inflorescence between species. Further RNA-seq experiments are needed to test this hypothesis.

## Figures and Tables

**Table 1:** Fifteen candidate genes related to flowering time differences identified in *Melastoma*. These genes contain one or more highly differentiated SNPs between two extreme bulks for flowering time in an F2 population between *Melastoma candidum* and *M. normale* and are orthologous to flowering time-related genes in *Arabidopsis thaliana.*

Gene_ID	Chromosome	Gene Name	Number of Highly Differentiated SNPs	Fst of Highly Differentiated SNP(s)	*Arabidopsis* Orthologs	Pathways of Flowering Time in *Arabidopsis* to Which the Gene Belongs
MLD38_026337	7	*CSTF77*	5	0.30–0.36	AT1G17760	-
MLD38_024073	7	*FY*	2	0.33, 0.35	AT5G13480	-
MLD38_027099	7	*SPA3*	1	0.31	AT1G53090, AT3G15354	-
MLD38_026174	7	*CDF3*	2	0.38, 0.43	AT3G47500, AT5G62430	Photoperiod pathway
MLD38_026194	7	*AGL8*	1	0.47	AT5G60910	Age pathway
MLD38_024433	7	*FUS3*	9	0.31–0.40	AT3G26790	-
MLD38_024058	7	*AGL15*	1	0.35	AT5G13790	-
MLD38_026516	7	*FHY1*	2	0.31, 0.32	AT2G37678, AT5G02200	-
MLD38_025118	7	*EZA1*	1	0.32	AT1G02580, AT4G02020	-
MLD38_026475	7	*COL9*	6	0.33–0.41	AT3G07650, AT5G48250	
MLD38_027028	7	*CIB1*	2	0.30, 0.38	AT4G34530	Photoperiod pathway
MLD38_025534	7	*FKF1*	6	0.32–0.45	AT1G68050	Photoperiod pathway
MLD38_023961	7	*FAR1*	5	0.32–0.40	AT4G15090, AT4G19990	-
MLD38_025645	7	*PGM*	12	0.30–0.40	AT5G51820	-
MLD38_025363	7	*HAP2A*	4	0.32–0.34	AT5G12840	-

**Table 2:** Large inversions on chromosome 7 between *Melastoma candidum* and *M. normale*.

Start Position	End Position	Inversion Size (bp)	Flowering-Related Genes Contained within the Inversion
757,425	3,760,509	3,003,084	*FY*, *AGL15*, *FAR1*
6,342,111	19,027,720	12,685,609	*CSTF77*, *CDF3*, *AGL8*, *EZA1*, *COL9*, *PGMP*, *HAP2A*, *FKF1*
10,533,038	17,653,689	7,120,651	*AGL8*, *PGM*, *FKF1*
10,896,857	12,709,275	1,812,418	*FKF1*
11,415,949	14,960,373	3,544,424	*FKF1*

**Table 3:** Four candidate genes related to flower number per inflorescence difference identified in *Melastoma*. These genes contain one or more highly differentiated SNPs between two extreme bulks for flower number per inflorescence in an F2 population between *Melastoma candidum* and *M. normale* and are orthologous to inflorescence architecture-related genes in *Arabidopsis thaliana.*

Gene_ID	Chromosome	Gene Name	Number of Highly Differentiated SNPs	Fst of Highly Differentiated SNP(s)	*Arabidopsis* Orthologs
MLD38_040423	12	*JAG*	5	0.22–0.30	AT1G13400, AT1G68480
MLD38_004029	2	*PNF*	1	0.22	AT2G27990
MLD38_023819	7	*FIL*	1	0.26	AT2G45190
MLD38_035107	10	*LAS*	2	0.24, 0.24	AT1G55580

## Data Availability

All raw sequencing data used in this research have been deposited in a genome sequence archive (GSA, Beijing, China, https://ngdc.cncb.ac.cn/gsa/). The accession number of this project is PRJCA008154, and the accession numbers of raw Illumina data are CRA005999, CRX327799, CRX327798, CRX327797 and CRX327796.

## Supplementary Materials

The following supporting information can be downloaded at https://www.mdpi.com/article/10.3390/ijms251910250/s1.
